# Size-Dependent Structural Properties of a High-Nb TiAl Alloy Powder

**DOI:** 10.3390/ma13010161

**Published:** 2020-01-01

**Authors:** Binglin Liu, Maosong Wang, Yulei Du, Jingxiao Li

**Affiliations:** 1 School of Mechanical Engineering, Nanjing University of Science and Technology, Nanjing 210094, China; 2 School of Mechanical Engineering, Nanjing Institute of Industry Technology, Nanjing 210016, China

**Keywords:** high-Nb TiAl, gas-atomized alloy powders, phase transition, size-dependent structural properties

## Abstract

TiAl-based alloys are promising light weight structural materials for high temperature applications in the field of aerospace. Recently, fabrication technologies starting from powders including powder metallurgy and additive manufacturing have been developed to overcome the difficulties in the processing, machining and shaping of TiAl-based alloys. Spherical alloy powders with different particle size distributions are usually used in these fabrication techniques. The purpose of this study is to reveal the size-dependent structural properties of a high-Nb TiAl powder for these fabrication technologies starting from powders. A high-Nb TiAl pre-alloyed powder with nominal composition of Ti-48Al-2Cr-8Nb (at. %) was prepared by the electrode induction melting gas atomization (EIGA) method. The phase structure and morphology of the as-atomized powders were characterized by X-ray diffraction (XRD) and scanning electron microscopy (SEM). The size-dependent structural changes of the as-atomized powders with different sizes were studied by differential scanning calorimetry (DSC) and in situ high temperature XRD. It was found that with decreasing the powder size, the content of the γ-TiAl phase decreases and the α_2_-Ti_3_Al phase increases. The α_2_-Ti_3_Al to γ-TiAl phase transformation was found in the temperature range of 600–770 °C. Based on the present work, the structural characteristics of TiAl powders are strongly dependent on their particle size, which should be considered in optimizing the process parameters of TiAl alloys fabricated from powders.

## 1. Introduction

TiAl-based alloys have been considered as promising structural materials for aerospace applications due to their light-weight and excellent high temperature mechanical properties. With increasing Nb content (usually > 5%), the service temperature of TiAl-based alloys can be further increased, which makes them more attractive for applications in gas turbine engines for replacing some Ni-based superalloys [1,2,3,4,5]. However, TiAl-based alloys are very brittle at room temperature, leading to difficulties in machining and forming. In recent years, in order to overcome the difficulties in the manufacturing of TiAl-based alloy parts, some fabrication technologies starting from powders have been developed. For example, powder metallurgy technology [6], in particular spark plasma sintering (SPS), was adopted to prepare TiAl-based alloy parts. It was found that the full densification can be achieved at a relatively lower temperature by using pre-alloyed TiAl powders with small size and disorder structures [7]. Bulk nanostructured Ti-45Al-8Nb alloy with ultrahigh compressive strength can be obtained via SPS by using cryo-milled elemental powders [8]. It is seen that the state of the original powders have great influence on the sintering behavior, microstructure and mechanical properties of the TiAl-based alloys fabricated by SPS. Lately, additive manufacturing (AM) technologies including laser/electron AM methods have been used to fabricate TiAl-based alloy parts. It is known that the different AM processes have different requirements for the particle size range of the powder used. For example, in the laser-engineered net shaping (LENS) process, TiAl-based alloy powders with a particle size between 45 and 180 µm are used [9]. While the powders with a size of 20 to 60 µm are used in the selective laser melting process [10] and the powders with an average size of 100 µm are used in the electron beam melting process [11]. Obviously, both of these fabrication technologies use powders as raw materials. Thus, the structural properties of the used TiAl-based alloy powders will definitely affect their fabrication processes. For example, based on the fundamentals of SPS, the sintering and densification behavior of TiAl-based alloy powders during the SPS process is strongly related to the state of the used powders [12]. Furthermore, the melting and solidification behavior of the TiAl-based alloy in the laser/electron additive manufacturing process is also affected by the size and size distribution, shape, and structure of the used powders [13,14]. Therefore, in order to optimize the fabrication process parameters of a TiAl-based alloy, it is critical to uncover the size-dependent structural properties of TiAl-based alloy powders. In the previous reports, the structural properties of some TiAl binary alloy powders and one low-Nb TiAl alloy powder have been studied [15,16,17]. The phase structure was found to be greatly size-dependent. In view of the fact that the structural properties of TiAl-based alloys are sensitive to their chemical composition and fabrication methods, and the promising applications of high-Nb TiAl alloys, it is very important to reveal the size-dependent structural properties of high-Nb TiAl alloy powders.

In this study, a high-Nb TiAl powder with a nominal composition of Ti-48Al-2Cr-8Nb (at. %) was prepared. The effect of particle size on the phase structure of the as-prepared high-Nb TiAl powders were revealed and the phase transition during heating of the as-prepared powders with different particle sizes were studied by in situ high-temperature XRD tests. The present results were valuable for optimizing the processing parameters of high-Nb TiAl alloy parts made from powders.

## 2. Materials and Methods

The high-Nb TiAl powder was produced by the electrode induction melting gas atomization (EIGA) method as follows: Firstly, the alloy ingots were made by cold crucible induction levitation melting of pure Ti, Al, Cr and Nb with purity above 99.9% under argon atmosphere. Then, the alloy rod with a diameter of 40 mm and a length of 400 mm was prepared by the metal mold casting method under a high-purity argon atmosphere. Finally, the as-casted rod dips into a conical induction coil and the tip of the rod melts. The droplets pour through the nozzle and are atomized by argon gas. The as-prepared powders are spherical in shape with a diameter ranging from several microns to more than 100 µm. The powder was collected and sieved into six different batches: 0–15 µm, 26–45 µm, 45–58 µm, 58–75 µm, 75–100 µm and 100–150 µm.

The phase structure of the as-prepared powders with different diameters was revealed by X-ray diffraction (XRD, D8 Advance, Bruker, Leipzig, Germany) with Cu-Kα radiation (λ = 1.5418 Å) at 30 kV and 10 mA. The structural evolution with temperature of the as-prepared powders was investigated by in-situ high temperature XRD experiments, in which the powders were put on a hot stage to be heated to the selected temperatures in a vacuum with a heating rate of 20 K/min, and then kept at the selected temperature for 10 min before the XRD measurements. The morphologies of the as-atomized powders with different diameters without any prior treatment were investigated by scanning electron microscopy (SEM, Quant250FEG, FEI, Hillsboro, OR., USA). The cross-sectional morphologies of the powders were observed by optical microscopy (OM, BX53M, OLMPUS, Tokyo, Japan), in which the powders were mounted in epoxy, polished and then etched by Kroll reagent (5% HF + 15% HNO_3_ + H_2_O). The phase transition during heating was also studied by differential scanning calorimetry (DSC, Netzsch STA 449C, Selb, Germany) at a heating rate of 20 K/min in a high purity argon atmosphere.

## 3. Results and Discussion

**Figure 1:**
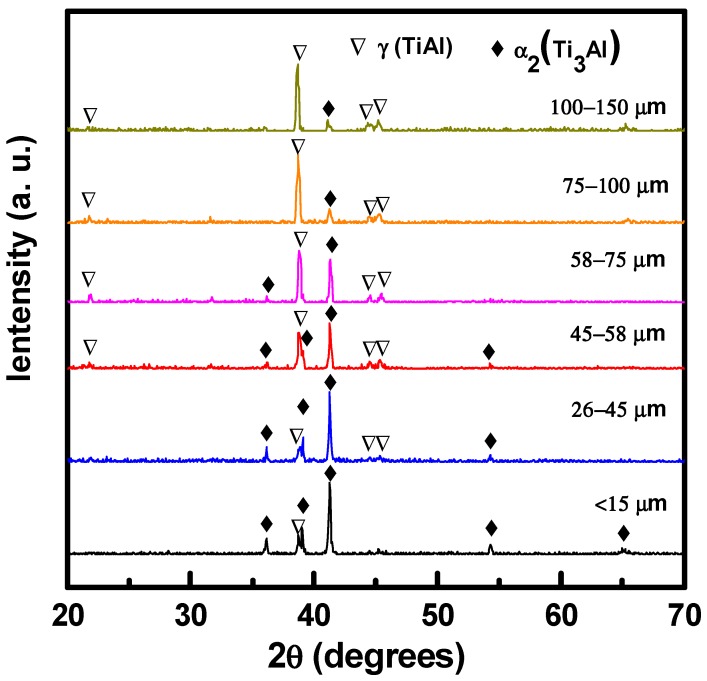
XRD patterns of the as-atomized high-Nb TiAl powders with different sizes.

Figure 1 shows the XRD patterns of the as-atomized high-Nb TiAl powders with different sizes at room temperature. It was observed that the phase structure of the as-atomized powders was strongly dependent on the particle sizes. The powders with a diameter larger than 100 µm are composed of a primary γ-TiAl (hereafter denoted as γ) phase and a small amount of α_2_-Ti_3_Al (hereafter denoted as α_2_) phase. With decreasing the powder size, the intensity of α_2_ peaks enhanced gradually, while that of γ peaks reduced gradually. The finest powder with a diameter less than 15 µm is dominated by the α_2_ phase, with only a minor fraction of the γ phase. As reported in the literature, the metastable α_2_ phase could easily form during rapid solidification [18,19]. Thus, in the present work, the appearance of the α_2_ phase in the high-Nb TiAl powders indicated that the cooling rate in the gas atomization process is rather high. For the fine powders, they undergo higher cooling rate during the solidification process than the larger powders, as a result, the amount of the α_2_ phase increases greatly. It should be noted that although Nb is a known β-stabilizer element, no β phase can be detected in the XRD patterns. This phenomenon indicates that the cooling rate is the key factor in determining the phase structure of high-Nb TiAl alloy powder during gas atomization. According to the XRD results, there is a size-driven γ to α_2_ structural transformation in the gas-atomized high-Nb TiAl powders.

**Figure 2:**
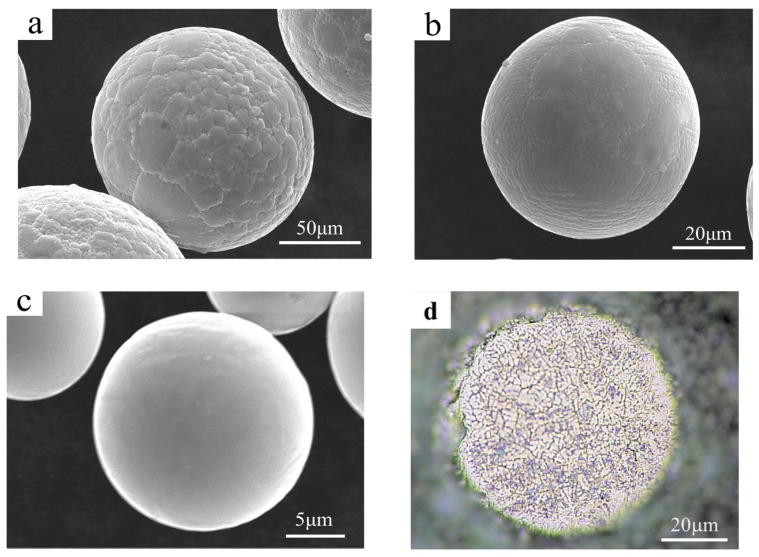
SEM surface morphology of the as-atomized high-Nb TiAl powder with different sizes: (**a**) large size (>100 µm); (**b**) medium size (45–75 µm); (**c**) fine size (<15 µm). (**d**) The cross-sectional microstructure of the medium powder.

Figure 2a–c shows the SEM surface morphology of the as-atomized high-Nb TiAl powders with large, medium and fine sizes, respectively. Three kinds of surface microstructures in the as-atomized powders can be observed. The powder with a diameter larger than 100 µm exhibits cellular structure. The powders with medium sizes (45–75 µm) demonstrate a nearly equiaxed dendrite structure with an obvious hexagonal or polygon surface pattern, while the fine powder (smaller than 15 µm) shows smooth and featureless surface morphology. Figure 2d shows the cross-sectional microstructure of the powder with a medium size. The equiaxed dendrite morphology can be clearly seen, which is consistent with its surface morphology. This size-dependent change in surface morphology could also be attributed to the different thermal gradient in the solidification process of the powders with different sizes [17,20]. According to the classical solidification theory, under sufficient high thermal gradient (in the case of the fine powder), the solidification with a plane front can be obtained, resulting in the smooth and featureless surface [21]. However, with increasing the particle size, the thermal gradient reduced, leading to the origination of the dendrite and cellular solidification mode. The smooth surface can improve the flowability of the fine high-Nb TiAl alloy powders, which is conducive to the stable running of the additive manufacturing process.

**Figure 3:**
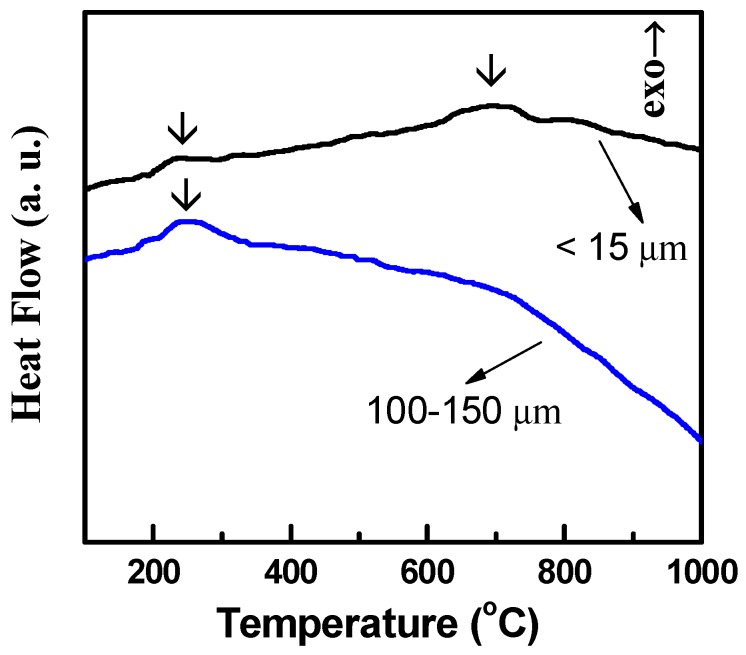
DSC (differential scanning calorimetry) curves of the as-atomized high-Nb TiAl powders with different sizes.

Based on the above results, it is seen that the fine high-Nb TiAl powder predominantly comprised the α_2_ phase, while the very coarse powder mainly comprised the γ phase. To further reveal the structural changes of the high-Nb TiAl powders with different sizes, thermal analysis was performed. Figure 3 exhibits the DSC curves for the as-atomized high-Nb TiAl powders with a diameter larger than 100 µm and less than 15 µm, respectively. The DSC curves of these two samples show an exothermic peak in the range of 200–290 °C, which can be attributed to the stabilization of the metastable α_2_ phase in the powder. Because of the rapid solidification during the gas atomization, many metastable structures, such as nanocrystalline, disorder structures, may form in the as-prepared high-Nb TiAl powders. When heating, these metastable structures transform to ordered stable structures, leading to this exothermic peak. However, this conjecture needs further experimental confirmation. For the fine (less than 15 µm) high-Nb TiAl powder with the α_2_ phase, there is an endothermic peak in the temperature range of 600–770 °C, which is related to the α_2_ to γ phase transition. It should be noted that for the coarse powder with the γ phase, this endothermic peak is absent, which also shows that this endothermic peak is due to the α_2_ to γ phase transition.

**Figure 4:**
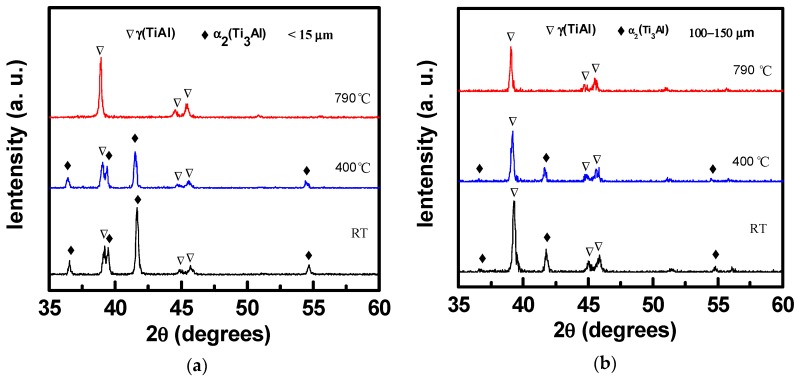
In situ high-temperature XRD patterns of the as-atomized high-Nb TiAl powders with different sizes: (**a**) fine size (<15 µm); (**b**) large size (100–150 µm).

In situ high temperature XRD (HT-XRD) is a powerful technique for the observation of a phase transition during heating. In the present work, the HT-XRD was conducted to characterize the structural transformation of the as-atomized fine and coarse high-Nb TiAl powders. According to the DSC curves, we selected two temperatures of 400 °C and 790 °C to determine the phase changes after the two exothermic peaks. It can be seen from Figure 4 that when heating to 400 °C, there were nearly no changes in the phase structure for both the fine and coarse powders, which indicates that the first exothermic peak at 200–290 °C in DSC curves is mostly caused by the stabilization of the metastable α_2_ phase, not by the phase transition in the rapid solidified powders. For the fine powder, with increasing the temperature to 790 °C, all the peaks belonging to α_2_ phase disappeared and only the peaks of the γ phase existed, which definitely implies that the second exothermic peak at 600–770 °C in the DSC curve is related to the α_2_ to γ phase transition. For the coarse powder, the main phase is γ at all the temperatures. The small amount of α_2_ phase in the coarse powder also disappeared at 790 °C. It is noted that with increasing temperature, all the peaks shift downwards, which can be attributed to the thermal expansion of the crystal lattice with increasing temperature.

Based on the present work, the phase structure of the as-atomized high-Nb TiAl alloy powder is significantly size-dependent. The amount of the metastable α_2_ phase increases as the particle size decreases. The powders with a particle size less than 45 µm primarily exhibit the α_2_ phase. It is known that in the selective laser melting additive manufacturing process, the fine powders with a particle size of 10–45 µm are usually used to print parts with high precision, while the relative coarse powders with a particle size of 45–100 µm are used to produce parts with low precision but high productivity. According to the present results, the fine and coarse high-Nb TiAl alloy powders have different phase structure and undergo different phase transition behavior when heated. In addition, it is expected that the fine and coarse high-Nb TiAl powders will show different densification behavior during the sintering process of SPS. Therefore, the size-dependent structural properties of high-Nb TiAl powders should be considered when optimizing the SLM and SPS process parameters.

## 4. Conclusions

In conclusion, the structural evolution of a high-Nb TiAl powder (Ti-48Al-2Cr-8Nb (at. %)) with particle sizes was revealed in the present work. The phase composition of the as-atomized high-Nb TiAl powder is largely dependent on the particle size. The introduction of high Nb has little effect on this size-dependent structural transformation. As the powder size reduces from a large size (100–150 µm) to a fine size (<15 µm), the surface morphology changes from cellular structure to equiaxed dendrite and finally to smooth structure. The finest powders crystallize in the metastable α_2_ phase due to the rapid solidification of gas atomization and undergo a α_2_ to γ phase transition in the temperature range of 600–770 °C. Based on the present work, the structural characteristics of high-Nb TiAl powders are strongly dependent on their particle size, which should be considered in optimizing the process parameters of TiAl alloys fabricated from powders.
